# Sendai virus is robust and consistent in delivering genes into human pancreatic cancer cells

**DOI:** 10.1016/j.heliyon.2024.e27221

**Published:** 2024-02-28

**Authors:** Dmytro Grygoryev, Taelor Ekstrom, Elise Manalo, Jason M. Link, Amani Alshaikh, Dove Keith, Brittany L. Allen-Petersen, Brett Sheppard, Terry Morgan, Abdenour Soufi, Rosalie C. Sears, Jungsun Kim

**Affiliations:** a Cancer Early Detection Advanced Research Center at Knight Cancer Institute, Oregon Health & Science University School of Medicine, USA; b Department of Molecular and Medical Genetics, Oregon Health & Science University School of Medicine, USA; c Brenden-Colson Center for Pancreatic Care, Oregon Health & Science University School of Medicine, USA; d The University of Edinburgh, Centre for Regenerative Medicine, Institute of Regeneration and Repair, Institute of Stem Cell Research, Edinburgh, UK; e King Abdulaziz City for Science and Technology, Health Sector (KACST), Riyadh, Saudi Arabia; f Department of Surgery, Oregon Health & Science University School of Medicine, USA; g Department of Pathology, Oregon Health & Science University School of Medicine, USA; h Cancer Biology Research Program, Knight Cancer Institute, Oregon Health & Science University School of Medicine, Portland, OR, 97201, USA

**Keywords:** Sendai virus (SeV), Pancreatic cancer, Gene delivery

## Abstract

**Background:**

Pancreatic ductal adenocarcinoma (PDAC) is a highly intratumorally heterogeneous disease that includes several subtypes and is highly plastic. Effective gene delivery to all PDAC cells is essential for modulating gene expression and identifying potential gene-based therapeutic targets in PDAC. Most current gene delivery systems for pancreatic cells are optimized for islet or acinar cells. Lentiviral vectors are the current main gene delivery vectors for PDAC, but their transduction efficiencies vary depending on pancreatic cell type, and are especially poor for the classical subtype of PDAC cells from both primary tumors and cell lines.

**Methods:**

We systemically compare transduction efficiencies of glycoprotein G of vesicular stomatitis virus (VSV-G)-pseudotyped lentiviral and Sendai viral vectors in human normal pancreatic ductal and PDAC cells.

**Results:**

We find that the Sendai viral vector gives the most robust gene delivery efficiency regardless of PDAC cell type. Therefore, we propose using Sendai viral vectors to transduce ectopic genes into PDAC cells.

## Highlights

• Systemic comparison of gene delivery methods for human pancreatic ductal adenocarcinoma.
• Lentiviral transduction is highly variable among human pancreatic ductal adenocarcinoma.
• Sendai virus gives the most robust gene delivery efficiency regardless of PDAC cell types.

## 1 Introduction

Pancreatic ductal adenocarcinoma (PDAC) is the most common type of pancreatic cancer. Despite extensive efforts toward developing treatments, the outcome of patients with PDAC remains poor, having the lowest 5-year survival rate among all cancers from 2010 through 2016 [1]. This dismal prognosis is partly due to a lack of early symptoms and noninvasive markers that allow for early diagnosis and provide the window of treatment opportunity [2,3]. Therefore, a better understanding of PDAC and identifying new potential therapeutic targets is essential to control this dismal disease.

Human PDAC is highly intratumorally heterogeneous and can be sub-classified into “classical” and “basal” (or “squamous")-like subtypes [[4], [5], [6], [7], [8], [9]] based on transcriptional and histological profiles [[4], [5], [6], [7]]. Global epigenetic and enhancer remodeling underlies subtype-specification [[10], [11], [12], [13]], and only a limited number of genomic alterations, such as *TP53* mutation and *MYC* or *KRAS* amplifications, are associated with the basal subtype [[6], [7], [8],^14^]. Both classical and basal subtypes can coexist in the primary tumors of a patient [[7], [8], [9]] and be plasticity depending on culture conditions [15], underscoring the importance of studying and manipulating both subtypes to better understand PDAC.

Moloney murine leukemia virus (MLV)-derived recombinant retroviral [16] and glycoproteins (G proteins) of vesicular stomatitis virus (VSV-G) pseudotyped recombinant human immunodeficiency virus lentiviral (LV) [17,18] vectors have been widely used for delivering transgenes into a mammalian host cell. However, these viral vectors integrate into the host genome.

Replication-deficient recombinant DNA virus-derived vectors, including adenoviral (AdVs) [19,20] and adeno-associated viral (AAV) [[21], [22], [23]] vectors, are leading platforms for delivering transgenes for gene therapy. AAV vector is an especially attractive gene delivery vector for in vivo gene therapy due to the controllable capsids that can be tailored depending on target cells [24]. For example, AAV2 is the prototype for the AAV family and is the most extensively studied serotype of AAV [25], but AAV6, 8 and 9 are more commonly used to transduce murine pancreatic beta and ductal cells [26,27], whereas AAV 5 is also used to transduce pancreatic cells, including PDAC [28,29]. However, AAV vectors have several shortcomings that remain to be overcome, including requiring a high dose of vector to obtain clinical outcomes and small genomic packaging capacities [24].

Sendai virus (SeV), a paramyxovirus known as the hemagglutinating virus of Japan (HVJ), is an enveloped virus with an RNA genome [30]. SeV replicates in the cytoplasm of host cells without DNA intermediates and no nuclear phase in its lifecycle. Thus, SeV does not integrate into the host genome [30]. SeV is safe for humans because it has never been linked to human disease [30]. Recombinant SeV vectors faithfully mimic the properties of natural SeV [[31], [32], [33], [34], [35]]. Because SeV uses the ubiquitous sialic acid as the cellular receptor, it has a broad cellular tropism and readily infects many tissues and cell types [[31], [32], [33], [34], [35]]. Fusion protein (F)-deficient SeV (SeV/ΔF) has been generated and demonstrated to be non-self-transmissible for gene therapy [31,36]. Therefore, the SeV vector has been widely used for various purposes, including cellular reprogramming [37,38].

Effective gene delivery is essential to modulate gene expression and identify potential gene-based therapeutic targets, which is particularly important for PDAC because it contains heterogeneous cell types and has different subtypes.

Viral vectors, including VSV-G-pseudotyped LV vectors [[39], [40], [41], [42], [43], [44]], AAV vectors [22,23,26,27], and AdV [22,[45], [46], [47], [48], [49]], have been used to deliver genes into normal pancreatic islet and acinar cells. While AdVs have shown robust gene delivery for pancreatic cells in vivo, it is a transient gene expression and a large number of viral particles are required for efficient transduction, which is associated with increased cytotoxicity [46]. Conditionally replicative AdVs under the control of pancreatic cancer-specific promoters that allow their progeny virus to infect neighboring cells are used to target PDAC for oncolytic therapy purposes [50,51]. However, conditionally replicative AdVs for oncolytic therapy are unsuitable for gene delivery since they transduce and lyse cancer cells.

AAV vectors [27] have been used for gene delivery in the murine PDAC model, and LV and AAV vectors have been used for gene delivery into human PDAC cell lines [29,52]. The LV vector is shown to provide the most efficient transduction in pancreatic exocrine cells in vitro for long-term gene expression [44]. However, the LV vector is mainly used to target pancreatic acinar or islet cells [[39], [40], [41], [42], [43]], and pancreatic ductal cells are resistant to LV transduction compared to other pancreatic cell types [44,53]. Some PDAC cell lines are highly resistant to the attachment and transduction of VSV, in which surface G-proteins are used to pseudotype LV for the highest transduction efficiency [[54], [55], [56]]. Moreover, we found that classical subtypes in both primary tumors and cell lines are particularly resistant to being transduced by lentivirus.

In this study, we aim to determine the gene delivery method that can achieve long-term gene expression of normal pancreatic ductal and classical and basal-subtype PDAC cells consistently with a relatively small amount of virus, which is required for CRISPR-mediated interference or cell state changes. Thus, we compare the transduction efficiencies of VSV-G-pseudotyped LV, SeV, and AAV vectors using classical and basal subtypes of PDAC cell lines as well as primary PDAC cells prepared from patient-derived xenograft (PDX) along with control cells by assessing the expression of a GFP reporter. We found that the SeV-mediated gene delivery system gave the most robust efficiency regardless of the tested PDAC cell types.

## 2 Materials and methods

### 2.1 Cell lines

Human Pancreatic Duct Epithelial (HPDE) cell line H6c7 (Kerafast, Boston, MA # ECA001-FP) was maintained in Keratinocyte SFM medium (Thermo Fisher Scientific, Waltham, MA. # 17005042). Human fibrosarcoma cell line HT1080 (ATCC # CCL-121; American Type Culture Collection (ATCC), Rockville, MD), human kidney cell line 293T (ATCC # CRL-3216), human pancreatic ductal adenocarcinoma (PDAC) cell lines Panc-1 (ATCC # CRL-1469) and MIA PaCa-2 (ATCC # CRM-CRL-1420) were maintained in Dulbecco's modified Eagle's medium (DMEM, Thermo Fisher Scientific # 10569010) supplemented with 10% fetal bovine serum (FBS). Human foreskin fibroblast cell line BJ6 (ATCC # CRL-2522) and PDAC cell line HPAF-II (ATCC # CRL-1997) were maintained in Eagle's minimum essential medium (EMEM ATCC # 30–2003) supplemented with 10% FBS. Human PDAC cell lines CFPAC-1 (ATCC # CRL-1918) and Capan-1 (ATCC # HTB-79) were maintained in Iscove's modified Dulbecco's medium (IMDM ATCC # 30–2005) supplemented with 10% and 20% FBS, respectively. Human PDAC cell line BxPC-3 (ATCC # CRL-1687) was maintained in RPMI-1640 medium (ATCC # 30–2001) supplemented with 10% FBS. Human PDAC cell line SW1990 (ATCC # CRL-2172) was maintained in Leibovitz's L-15 medium (ATCC # 30–2008) supplemented with 10% FBS. All cell lines except SW1990 were incubated at 37 °C in a humidified incubator with 5% CO_2_. SW1990 cell line was incubated at 37 °C in a humidified incubator. Mycoplasma test was routinely performed every three months in each cell line.

### 2.2 Establish patient-derived xenograft (PDX) primary tumor cell culture

Human PDAC specimens were obtained under the Oregon Pancreas Tissue Registry study (IRB00003609). Informed consent was obtained from all subjects. All experimental protocols were approved by the OHSU Institutional Review Board. All methods were carried out in accordance with relevant guidelines and regulations. All animal works for PDX tumors were performed with the OHSU Institutional Animal Use and Care Committee (IACUC) approval. PDX tumors were generated by implanting primary PDAC tissue in NOD-*Scid* IL2Rgamma^null^ (NSG) mice and were collected after tumor size surpassed 1 cm^2^ in diameter. Tumors were dissociated using human tumor dissociation kit (Miltenyi Biotec #130-095-929) by the Miltenyi Biotec gentleMacs Octo Dissociator (#130-096-427) with program 37_h_TDK_2 for 1hr. Mouse cells were removed from dissociated tumor cells to enrich human cells using mouse cell depletion (Mouse Cell Depletion Kit, Miltenyi Biotec # 130-104-694). Samples were taken before and after mouse depletion to assess human cell enrichment via flow cytometry (FACSymphony™ A5 SE Cell Analyzer, BD Biosciences, San Jose, CA). Cells were stained with BV421 Mouse Anti-Human TRA-1-85 Antigen (BD Biosciences, #563302) and live-dead cell Fixable Viability Stain 660 (BD Biosciences, # BDB564405) prior to the run. Samples were gated for size and viability before quantifying human TRA-1-85 cells among live cells.

To establish primary cell cultures with PDX PDAC (ST-7599, ST-14490, ST-7270 and ST-12908), dissociated human cell enriched PDX-PDAC cells were seed with density 1 × 10^6^ cell/well of 6-well plates coated with 10 μg/cm^2^ of rat tail collagen-I (Corning, Corning, NY # 354,236). The cells were maintained in completed PDAC media as previously described [[57], [58], [59]] (Completed Defined K-SFM medium (Thermo Fisher Scientific #10744–019) supplemented with 5 ng/ml human EGF (BD Bioscience #354052), 50 ng/ml cholera toxin (Sigma, St. Louis, MO #C8052) and 50 μg/ml bovine pituitary extract (Thermo Fisher Scientific, #13028014) under physiological conditions (37 °C, 5% O₂, 5% CO₂).

### 2.3 LV-GFP, SeV-GFP, and AAV-GFP viral production

LV-GFP vector was produced as described previously [57]. Briefly, 8 × 10^5^ of 293T cells were transfected 24 h after plating with 2.5 μg vector PWPT-GFP, 1.7 μg psPAX2 packaging vector, and 0.8 μg pMD.G envelope vector with 30 μl Fugene 6 (Promega, Madison, WI #E2691). The virus supernatant was collected 76 h post-transduction and concentrated by ultra-centrifugation (Beckman Coulter SW 32 Ti rotor, 25,000 rpm for 1 h 30 min).

SeV-GFP viruses were purchased at Thermo Fisher Scientific (CytoTune™ EmGFP Sendai Fluorescence Reporter, # A16519). AAV-GFP (AAV2 and AAV5) viruses were prepared by the OHSU Molecular Virology Core at the Oregon National Primate Research Center.

### 2.4 Transduction, determination of vector titration, and relative transduction efficiencies

Cells were transduced with serially diluted viral vectors (10^−2^ to 10^−6^) with 4.5 μg/mL polybrene (Sigma, # TR-1003-G) 24 h post-plating with a density of 1 × 10^5^ cells/4 cm^2^, and medium was replaced with fresh medium 24 h after transduction. Mock-transduced cells were used as negative controls. 72 h after transduction, cells were imaged, stained with live-dead marker Fixable Viability Stain 660 (BD Biosciences, # BDB564405), collected, fixed with 4% PFA for 30 min, washed with PBS, and analyzed for GFP expression by flow cytometry. Ten thousand events per sample were gated for size, followed by viability, before measuring the fraction of GFP-positive cells out of the total live cells.

Functional titers of LV-GFP and SeV-GFP vectors were determined on HT1080, BJ6, H6c7, Panc-1, CFPAC-1, SW1990, BxPc3, Capan-1, MIA PaCa-2, HPAF-II, ST-7599, ST-14490 and ST-7270 cells with GFP expression level by flow cytometry (FACSymphony™ A5 SE Cell Analyzer, BD Biosciences)**.** Cell-specific functional titers were calculated as follows: functional infectious units per milliliter (IFU/ml) = (Cell number at the time of transduction x percentage of GFP positive cells)/(Virus volume in mL). For AAV vectors, titration was conducted only on H6c7 cells, and polybrene was not added to the medium.

To obtain the dependence of the transduced cell fraction on the amount of viral vectors IFU/cell, we transduced cells with different amounts of viral vectors (calculated based on previously determined titers) in a 0.5 ml volume of culture medium with 4.5 μg/mL polybrene (Sigma, #TR-1003-G). Mock-transduced cells were used as negative controls. GFP level was analyzed 72 h post-transduction as described above.

### 2.5 Cell nucleofection

BJ6, H6c7, Panc-1, CFPAC-1, SW1990, BxPc3, Capan-1, MIA PaCa-2, ST-7599 and ST-12908 cells were transfected with GFP-containing plasmids using a 4D-Nucleofector X Unit (Lonza Inc., Morristown, NJ) with SE Cell Line Kit S (Lonza Inc., #V4XC-1032) in a 20 μL format according to the manufacturer's protocol. Primary PDAC cells (ST-7599 and ST-12908) were transfected using the Primary Cell Optimization Kit (Lonza Inc., #V4XP-9096) according to the manufacturer's protocol. The nucleofection programs and the nucleofector solutions used for primary PDAC cells and cell lines are presented in Fig. S3C-F and Fig. S3G.

### 2.6 Bulk RNA-sequencing (RNA-seq) and qRT-PCR

To determine subtypes from primary PDAC cells or PDAC cell lines, we conducted RNA-Seq or qRT-PCR, respectively. Total RNAs were isolated from each cell line or primary cell using the RNeasy Mini Kit (Qiagen, Germantown MD, # 74,104). RNA-seq from primary PDAC cells was performed to target 60 million reads using a NovaSeq 6000 sequencer by Novogene. Reads with adapter sequences, reads with low quality (<Q20), short reads (<35bp), or reads with too many Ns (>5) were removed. Trimmed reads were aligned to the GRCh38. p13 using STAR [60]. RNA-seq raw counts were normalized with DESeq2 version 1.36.0 R package [61].

cDNAs from PDAC cell lines were synthesized with iscript Advanced cDNA Synthesis Kit (Bio-rad, Hercules, CA #1725037) for qRT-PCR. *GAPDH, PDX1, GATA6, MNX1, TP63, KRT5 and LY6D* gene expression levels were determined from PDAC cell line-derived cDNAs with Taqman™ Gene Expression Assays (Hs02786624_g1; Hs00232018_m1; Hs00232018_m1; Hs00907365_m1; Hs00186613_m1; Hs00361185_m1 and Hs00933261_g1, respectively) and TaqMan™ Fast Advanced Master Mix (Thermo Fisher Scientific #4444556) through QuantStudio 6 qPCR System (Thermo Fisher Scientific).

### 2.7 Characterizing transcriptional subtypes of primary PDAC cells

PDAC subtypes are identified based on transcriptional profiles and defined by a combination of markers [[4], [5], [6], [7], [8]]. Among them, GATA6 is a well-established classical subtype marker; Loss of GATA6 is linked to the transition to the basal phenotype [[62], [63], [64]]. The concomitant loss of GATA6, HNF1A, and HNF4A in PDAC is required for the expression of TP63 isoform deltaNp63, which is a basal marker, and a switch towards the basal subtype in patients and mice [62,63]. Accordingly, we verified PDAC subtypes with multiple clustering. The subtypes of PDAC cell lines (Panc-1, CFPAC-1, SW1990, BxPc3, Capan-1, MIA PaCa-2 and HPAF-II) were previously characterized [65]. To determine the subtypes of established primary PDAC cells (ST-7599, ST-14490, and ST-7270), hierarchical and consensus clustering were performed with normalized gene expression (Figs. S1A and B) using PDAC subtypes gene expression signatures [7]. Additional consensus clustering was computed using *GAPDH* normalized subtype marker genes (*PDX1, GATA6, MNX1* for classical subtype [6,7] and *TP63, KRT5 and LY6D* for basal subtype [66,67]) in primary PDAC cells (ST-7599, ST-14490 and ST-7270) along with PDAC cell lines (CFPAC-1, Capan-1, HPAF-II, SW1990 and BxPc3) (Fig. S1C).

### 2.8 Quantification and statistical analysis

All experiments were independently performed at least three times, except ST-7599 and ST-12908 primary PDAC cells for the nucleofection experiment, where one measurement for each sample and condition was made. The results are presented as mean ± standard deviation (SD).

RNA-seq raw counts were normalized with DESeq2 version 1.36.0 R package [61]. Hierarchical clustering was performed with Pheatmap version 1.0.12 R package [68] using Euclidean distance. Consensus clustering was performed with ConsensusClusterPlus v. 1.60.0 R package [69] using Euclidean distance with 10,000 re-samplings to generate the final consensus. Two main clusters were identified based on silhouette scores, the relative change between k values, and the proportion of ambiguous clustering scores. Regression models fitting and analysis of the statistical significance of categorical variables (such as cell type or viral vector type) interactions was performed with Stats version 4.2.1 R package [70]. The statistical significance of relative transduction efficiency differences was calculated using the nonparametric Mann-Whitney *U* test.

## 3 Results

### 3.1 Classical subtype PDAC cells are resistant to LV transductions

**Fig. 1:**
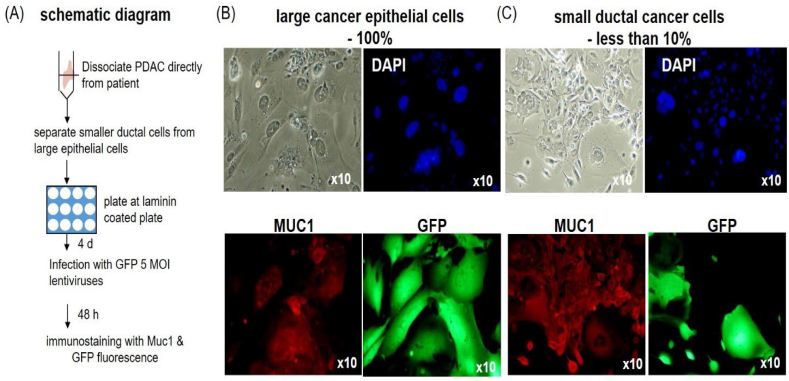
The heterogeneous susceptibility to LV transduction among human pancreatic cancer epithelial cells. (A) The schematic diagram for the transduction of primary human PDAC with LV-GFP. (B–C) GFP expression in epithelial cells derived from freshly isolated human PDAC cells. MUC1 is a marker for PDAC. While most larger epithelial cells express GFP (B), smaller ductal epithelial cells barely express GFP (C).

**Fig. 2:**
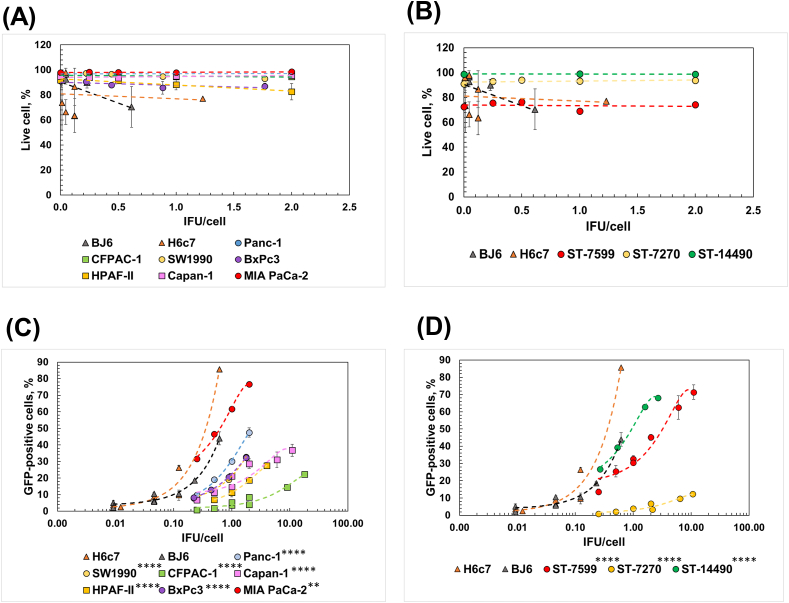
Transduction of human PDAC cells with LV-GFP. (A–B) Effect of LV-GFP transduction on the cell viability of PDAC cell lines (MIA PaCa-2, BxPC3, Panc-1, SW1990 (basal-like subtypes, circles) and CFPAC-1, Capan-1, and HPAF-II (classical subtypes, squares)) (A) and PDX-derived primary PDAC cells (ST-7599, ST-7270, and ST-14490) (B). Human normal fibroblasts (BJ6) and HPDE cells (H6c7) were used as controls. The percentage of live cells was calculated using D Horizon™ Fixable Viability Stain 660 by flow cytometry. Statistical significance was computed through linear regression model coefficients (p > 0.05, Table S2). The asterisks in the figure refer to the *P*-value of linear regression model coefficients. * - P ≤ 0.05; ** - P ≤ 0.01; *** - P ≤ 0.001; **** - P ≤ 0.0001. (*C*–D) Transduction efficiency of LV-GFP in PDAC cell lines (MIA PaCa-2, BxPC3, Panc-1, SW1990 (basal-like subtypes, circles) and CFPAC-1, Capan-1, and HPAF-II (classical subtypes, squares)) (C) and PDX-derived primary PDAC cells (ST-7599, ST-7270, and ST-14490) (D). BJ6 and H6c7 cells were used as controls. Functional infectious units per cell (IFU/cell) were calculated based on LV GFP titer in human fibrosarcoma HT1080 cells (Table S1). The dependence of the fraction of GFP-expressing cells to the number of viral IFU/cell were fitted with a second-degree polynomial regression model. Statistical significance was computed through ANOVA to test whether there were any significant interactions of categorical variables (cell types) in the regression models estimating the relationships of the percentage of GFP-expressing cells to the number of viral IFU/cell used (Table S3 for p-value). The asterisks in the figure refer to the *P*-value of the ANOVA test. * - P ≤ 0.05; ** - P ≤ 0.01; *** - P ≤ 0.001; **** - P ≤ 0.0001. Each experiment was repeated at least three times independently (n ≥ 3). All data are represented as mean ± SD unless specified. SD bars can be smaller than the size of the symbols.

We observed that the expression of LV vector-mediated transgenes in freshly prepared primary human PDAC cells varies depending on cell type (Fig. 1). This variation can be caused by heterogeneous cellular tropism of the LV vector (efficiency of gene delivery) or heterogeneous expression levels of the transgene. Thus, to avoid confounding effects caused by the variable expression through different promoter usages, we used a LV-vector expressing GFP under the human elongation factor 1α (EF-1a) promoter, which has fairly consistent activity across different cell types [71,72]. We first determined the functional titer of the virus [infectious unit (IFU)/ml] by counting the number of GFP-expressing cells by flow cytometry in HT1080 control cells transduced with a given amount of virus as described in the methods (Table S1). Then, to systemically examine the transduction efficiency in different subtypes of PDAC cells, we transduced across all cell types with a series of amounts of LV-GFP, expressed as functional infectious units (IFU/cell), and measured the fraction of cells expressing GFP 72 h after transduction (Fig. 2).

We then transduced four basal subtype PDAC lines (MIA Paca-2, BxPC3, Panc-1 and SW199) [65], three classical subtype PDAC lines (CFPAC-1, Capan-1 and HPAF-II) [65], and three primary PDAC cell cultures established from PDX (ST-7599, ST-7270 and ST-14490) as well as normal human Pancreatic Duct Epithelial Cell Line (HPDE) H6C7 cells, normal human foreskin fibroblast BJ6 cells, and human fibrosarcoma HT1080 cells. Primary PDAC subtypes were characterized by RNAseq and qRT-PCR (Fig. S1).

Delivering LV-GFP vectors with a wide IFU/cell range did not significantly impair the viability of any tested cells (*p* > *0.05*, Fig. 2A–B; Table S2 for linear regression model coefficients p-values), indicating that the transgene product did not cause unintended toxicity among different cell types. However, GFP expression levels in most PDAC cells were significantly lower than those in HPDE and BJ6 cells (*p*<*0.05*, Fig. 2C–D; Table S3 for ANOVA p-values). For example, GFP expression levels in MIA PaCa-2 and ST-14490 cells were comparable to those in BJ6 cells (*p* = *0.5*) but were significantly lower than in H6c7 HPDE cells (p = *9.13E-03* for MIA PaCa-2*, p* = *1.87E-08* for ST-14490, Table S3). Moreover, transduction efficiencies in the basal subtype PDAC cell lines (MIA PaCa-2, BxPC3, Panc-1 and SW1990) were significantly higher compared to those in the classical subtype PDAC cell lines (CFPAC-1, Capan-1 and HPAF-II) (Fig. 2C). In sum, the transduction efficiency of LV vectors varied among pancreatic cell types.

### 3.2 The SeV vector provides efficient delivery and expression of the transgene in various PDAC cells

**Fig. 3:**
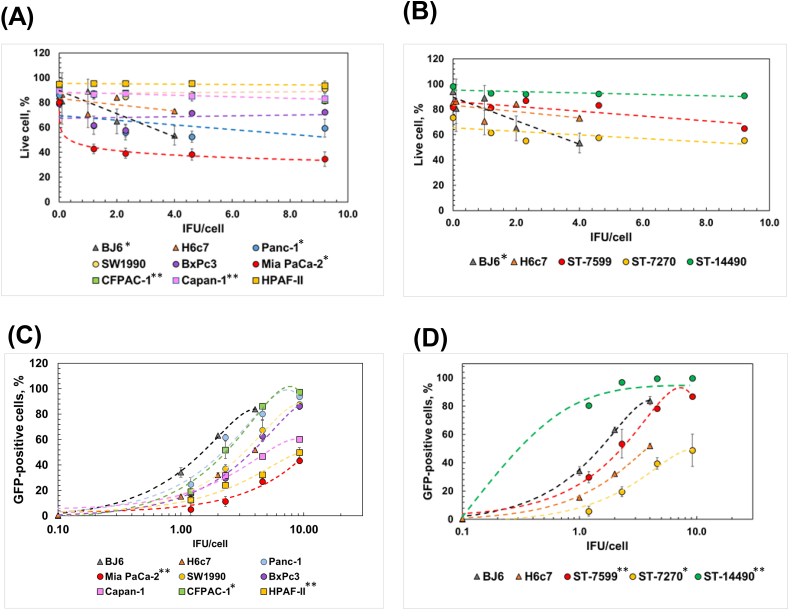
Transduction of human PDAC cells with SeV-GFP. (A–B) Effect on SeV-GFP transduction on the cell viability of PDAC cell lines (MIA PaCa-2, BxPC3, Panc-1, CFPAC-1, SW1990, Capan-1, and HPAF-II) (A) and PDX-derived primary PDAC cells (ST-7599, ST-7270, and ST-14490) (B). BJ6 fibroblast and H6c7 HPDE cells were used as normal controls. The percentage of live cells was calculated using D Horizon™ Fixable Viability Stain 660 by flow cytometry. Statistical significance was computed through linear regression model coefficients (Table S2 for p-values). (*C*–D) Transduction efficiency of SeV-GFP in PDAC cell lines (MIA Paca-2, BxPC3, Panc-1, CFPAC-1, SW1990, Capan-1, and HPAF-II) (C) and PDX-derived primary PDAC cells (ST-7599, ST-7270, and ST-14490) (D). As controls, BJ6 and H6c7 cells were used. Functional infectious units per cell (IFU/cell) were calculated based on SeV-GFP titer in LLC-MK2 cells (Table S1). The dependence of the fraction of GFP-expressing cells to the number of viral IFU/cell was fitted with a second-degree polynomial regression model. Statistical significance was computed through ANOVA to test whether there are any significant interactions of categorical variables (cell types) in the regression models estimating the relationships of the percentage of GFP-expressing cells to the number of viral IFU/cell used (Table S3 for p-value). The asterisks in the figure refer to the *P*-value of the ANOVA test. * - P ≤ 0.05; ** - P ≤ 0.01; *** - P ≤ 0.001; **** - P ≤ 0.0001. Each experiment was repeated at least three times independently (n ≥ 3). All data are represented as mean ± SD unless specified. SD bar can be smaller than the symbol's size.

We then tested the SeV vector using the same panel of cells. We first examined whether the amount of SeV affected cell viability through a linear regression model. We found that increasing amounts of SeV were not associated with the viability of primary PDAC cells but were correlated with decreased viability of a subset of PDAC cell lines (Fig. 3A–B, Table S2). The impact of SeV particles on cell viability was relatively mild, even in the highest amounts (over 60% and 40% cell viability for primary PDAC and PDAC cell lines, respectively, Fig. 3A–B, Table S2). Surprisingly, the transduction efficiencies of SeV-GFP in more than half of the tested PDAC cells were comparable to or higher than those in HPDE cells and BJ6 cells and were consistent regardless of tumor subtypes (Fig. 3C–D, Table S3 for ANOVA p-values).

### 3.3 SeV vector provides transduction efficiency comparable to or better than an LV vector in PDAC

The transduction efficiency of viruses varies depending on the cell type. Thus, to compare the transduction efficiencies of LV and SeV vectors in a given cell type, we measured functional viral titers (IFU/ml) of both viral vectors across all tested cells (Table S1). The number of viral vectors transduced per cell [functional infectious units per cell (IFU/cell)] for both viruses in each cell type was calculated based on their corresponding functional viral titers (Table S1).

**Fig. 4:**
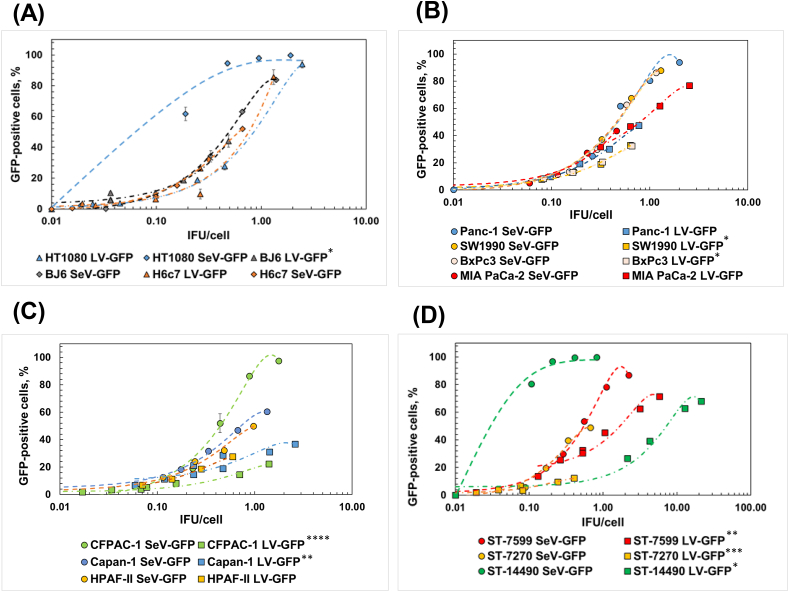
Comparison of transduction efficiencies between LV-GFP and SeV-GFP. Transduction efficiencies are compared in control cell lines (HT1080, BJ6, and H6c7) (A), classical subtype PDAC lines (CFPAC-1, Capan-1, and HPAF-II) (B), basal-like subtype PDAC lines (MIA Paca-2, BxPC3, Panc-1, and SW1990) (C), and PDX-derived primary PDAC cells (ST-7599, ST-7270, and ST-14490) (D). The IFU/cell is calculated based on LV-GFP and SeV-GFP titer in corresponding cells. Each experiment was repeated at least three times independently (n ≥ 3). A second-degree polynomial regression model fitted the dependence of the fraction of GFP-expressing cells on the number of viral IFU/cell used. Statistical significance was computed through ANOVA to test whether there are any significant interactions of categorical variables (viral vector type) in the regression models estimating the relationships of the percentage of GFP expressing cells to the number of viral IFU/cell used (Table S4 for p-value). The asterisks in the figure refer to the *P*-value of the ANOVA test. * - P ≤ 0.05; ** - P ≤ 0.01; *** - P ≤ 0.001; **** - P ≤ 0.0001. All data are represented as mean ± SD unless specified. An SD bar can be smaller than the symbol's size.

We then compared transduction efficiencies of SeV and LV in PDAC and normal cells by measuring the GFP expression level in transduced cells with a given IFU/cell value. The result showed that transduction efficiencies of LV-GFP and SeV-GFP were similar in H6C7 HPDE cells (*p* > *0.05*, Fig. 4A, Table S4 for ANOVA p-values). However, the transduction efficiency of SeV-GFP was significantly higher than that of LV-GFP in most basal and classical subtypes PDAC cell lines and all primary PDAC cells in a given IFU/cell value (*p*<*0.05*, Fig. 4B–D, Fig. S1, Table S4 for ANOVA p-values).

**Fig. 5:**
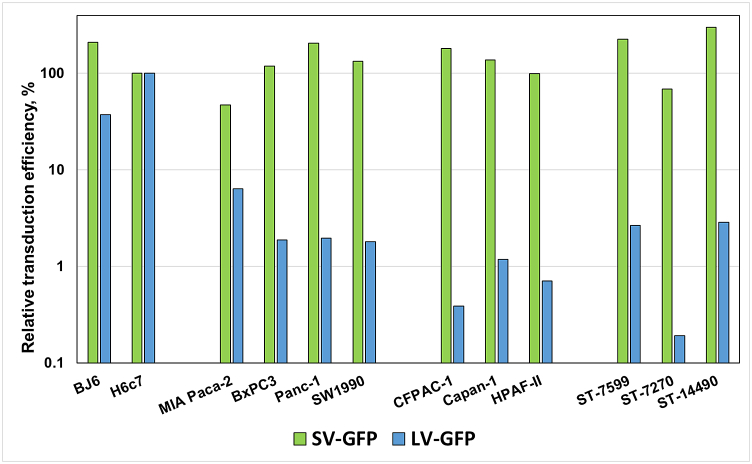
Relative transduction efficiencies of LV-GFP and SeV-GFP in PDAC cell lines and PDX-derived primary PDAC cells. The relative transduction efficiencies of LV and SeV vectors in PDAC cells (cell lines or primary cells) were calculated by normalizing titers obtained from PDAC cells to the titers obtained on the control H6c7 HPDE cells (n = 3). Statistical significance was computed with the nonparametric Mann-Whitney *U* test. The relative transduction efficiency of SeV-GFP was significantly higher than that of LV-GFP across all tested PDAC cells (Mann Whitney *U* test, *p*<*0.05*). The relative transduction efficiency of LV-GFP was significantly lower in the classical subtype than in the basal-like subtype of PDAC (Mann Whitney *U* test, *p* = *0.04*). In contrast, there was no difference in the relative transduction efficiencies of SeV-GFP between classical and basal-like subtypes of PDAC cells (Mann Whitney *U* test, *p* = *1*). Basal-like subtype PDAC lines (MIA Paca-2, BxPC3, Panc-1, and SW1990); classical subtype PDAC lines (CFPAC-1; Capan-1; HPAF-II; Primary PDAC cell (ST-7599; ST-7270; ST-14490); foreskin fibroblast BJ6; HPDE cell (H6c7). Since the presented data are not the result of direct measurements and do not satisfy all propagation of error requirements [81], the standard deviations are not shown.

To directly compare LV and SeV vectors, we computed relative transduction efficiencies of LV-GFP and SeV-GFP in PDAC cell lines as well as in primary PDAC cells (Fig. 5). For both vectors, the functional titers in PDAC cell lines and primary cells were normalized against the functional titer obtained using H6c7 cells, which was represented as 100. The result showed that SeV-GFP had significantly higher relative transduction efficiency than the LV vector in all tested PDAC cells (Fig. 5, Mann Whitney *U* test, *p*<*0.05*). No difference in relative transduction efficiency was observed between basal and classical subtypes of PDAC cells for SeV-GFP (Mann Whitney *U* test, *p* = *1*). In contrast, LV-GFP had significantly lower relative transduction efficiency in the “classical” subtypes of PDAC cells than in the basal subtype (Mann Whitney *U* test, *p* = *0.01*) (Fig. 5).

Together, these data show that SeV can transduce all tested PDAC cells with the ectopic gene more consistently and efficiently than the lentivirus.

### 3.4 AAV and non-viral methods do not provide a transduction efficiency better than LV or SeV

Since AAV is widely used for gene therapy and different AAV serotypes can be tailored to transduce certain cell types, we tested whether the AAV vector could provide a better transduction efficiency than the LV or SeV in HPDE cells. We tested two AAV serotypes, AAV2, a prototype serotype and best studied of the AAV serotypes, and AAV5, known to efficiently transduce pancreatic cells, including PDAC [28,29]. Although AAV9-based vectors are used widely, AAV 9 was excluded from the test since AAV 9 had low transduction efficiency in pancreatic ductal cells, especially comparing other pancreatic cell types, via either in vivo systemic or retrograde pancreatic ductal delivery [27], which is not suitable for heterogeneous primary PDAC cells.

Comparing models describing the dependencies of GFP-expressing cells to the number of IFU/cells in HPDE cells transduced with LV-GFP, SeV-GFP, AAV2-GFP, and AAV5-GFP showed that transduction efficiencies of both tested AAV serotypes were similar (*p-value* = *0.187*). However, the dependencies were significantly different (*p < 0.05*) in both AAV serotypes in the tested IFU/cell range from that of SeV-GFP vectors (Fig. S2). For example, in the 1 IFU/cell range, the percentage of GFP-expressing cells was lower for both AAV vectors compared to LV-GFP and SeV-GFP vectors.

Next, we tested whether non-viral transfection methods, such as nucleofection, would yield transduction efficiencies better than the SeV or LV vectors. Although a subset of nucleofector conditions resulted in good transfection efficiency in some PDAC cell lines (Figs. S3A–B), they were not better in primary PDAC cells (Figs. S3C–G).

In sum, neither tested AAV serotypes nor DNA transfection through nucleofection provided a better transduction efficiency than the LV or SeV vector in all tested normal pancreatic ductal cells or primary PDAC cells.

## 4 Discussion

Human PDAC is a heterogeneous disease that includes several subtypes. While targeting one cell type of interest would be useful, PDACs are also highly plastic. Therefore, robust and reproducible delivery of genes being studied across all cell types regardless of their states is essential in gene-based therapeutic approaches or in understanding the biological function of those genes in research for human PDAC. Herein, we systemically tested gene delivery methods for human PDAC cells and, for the first time, demonstrated that SeV most robustly delivers a genetic material across all PDAC cells, with efficiencies comparable to or higher than those in their normal counterpart HPDE or fibroblast cells. The advantages and utilities of SeV to the pancreatic field are described below.

First, SeV can robustly and consistently transduce human pancreatic ductal epithelial cells with genes regardless of whether they are normal or cancerous, primary or cell lines, or cancer subtypes. Human PDAC tissues consist of various normal cell types as well as benign and malignant neoplastic cells, and even malignant cells have a high degree of intra-tumoral heterogeneity and can be subtyped into classical and basal (or squamous) subtypes. Herein, we demonstrate SeV as the best gene delivery vector to efficiently transduce all tested human pancreatic and PDAC ductal cells.

The LV, AAV vector, and DNA transfection through nucleofection or traditional chemical methods are widely used to deliver genetic materials to be expressed or to perturb genetic functions in mammalian cells. However, it has been shown that some cancers, especially PDAC, are resistant to being transduced with LV [44]. Indeed, our study shows that the transduction efficiency of the LV vector varies depending on the cells and PDAC subtypes and is significantly lower in primary human PDAC cells than in normal cells or cancer cell lines. This high variability in LV transduction, such as “normal cell-biased transduction” and “uneven transduction among malignant cells”, would be a severe problem when genes are needed to be expressed in primary PDAC cells and would hamper our efforts to understand the outcome of loss or gain of genes or gene function. Neither tested AAV vector-mediated transduction nor DNA transfection through nucleofection could improve gene delivery efficiently to normal pancreatic ductal cells compared to LV or SeV.

Therefore, herein, we demonstrate SeV as the best gene delivery vector to efficiently transduce all tested human pancreatic and PDAC ductal cells.

Second, the SeV vector can effectively deliver transgenes without leaving a scar in the host genome since the SeV RNA genome replication occurs in the cytoplasm without a DNA phase [31]. DNA or RNA transfection or viral vectors such as adenovirus [19,20] and AAV [22,23,26,27] can also express transgenes without integrating into the host genome. Still, the expression of transgenes delivered by these methods is relatively unstable and transient in highly dividing cells such as cancer cells.

In contrast, although the SeV vector is an episomal RNA viral vector, we and others have found that transgenes delivered by the SeV vector remain in highly dividing host cells for up to five passages, enabling cells carrying transgenes for the periods sufficient to change cell states [37,38]. Therefore, the SeV vector can be an efficient RNA-based gene delivery vehicle for long-lasting overexpressing transgenes or perturbing endogenous genes in human PDAC and normal human pancreatic ductal cells to some extent for several passages.

Third, the SeV vector is also applicable for cell therapy, such as in vivo transdifferentiation or direct reprogramming in pancreatic diseases. For example, pancreatic acinar can be transdifferentiated into functional beta-cells using adenovirus expressing pancreatic master transcription factors, Ngn3, Pdx1, and Mafa, for managing diabetes [47]. Most beta cell transdifferentiation studies have been conducted in mouse models using adenovirus. Partial reprogramed PDAC using OCT4, SOX2, MYC, and KLF4 differentiated to reestablish a PDAC state from early to invasive stages or showed attenuated malignancy [57,73]. Recently, reprogramming cancer cells with master TFs like PU.1, IRF8, and BATF3 into dendritic cells or with CEBPA and PU.1 into macrophage-like cells demonstrated that the feasibility of restoring antigen-processing machinery and enhancing tumor-eradicating immunity [74,75].

Such transdifferentiation or direct reprogramming studies require a sustained, controlled polycistronic expression of multiple genes, underscoring the need for an effective gene delivery system in vivo. Yet, in vivo cell therapy is still in its early stages. Thus, to assess the clinical utility of these potential therapeutic approaches, safe vectors should be used to express genetic factors in the target cells of human tissues or non-human primate models.

Adenovirus and AAV are widely used for gene therapy but have limitations in cell therapy. Although adenovirus transduction is highly efficient in most cells and the onset of the expression of transgenes is rapid, adenovirus triggers a high immune response in the host. Moreover, high doses of adenovirus are highly toxic to the cells and the expression of transgenes delivered by adenovirus is transient. While AAV triggers very low levels of immune response [76], it has several drawbacks, such as integrating a small fraction of its genome into the host genome and later onset of transgene expression [77]. Especially, requiring a high dose of vector to achieve phenotypic outcomes and limited packing capacity for multiple transgenes in AAV [77] constrains its use in cell therapy applications, particularly when multiple master transcription factors need to be uniformly expressed.

Because primitive ductal epithelial layers give rise to the endocrine cells during development, and a significant portion of the pancreata comprises ductal cells, ductal cells could be an excellent candidate to initiate transdifferentiation into beta cells [78,79]. Since SeV robustly delivers genes into human pancreatic ductal cells without genome integration, delivering genetic factors into human pancreata using SeV would improve safety and efficacy in pre-clinical or clinical transdifferentiation studies for pancreatic diseases such as diabetes, pancreatitis, and PDAC. Moreover, SeV has never been linked to human disease [[31], [32], [33]]. Indeed, SeV is widely used to generate iPSCs for cell therapy purposes [37,38]; thus, the safety of SeV in cell therapy has been verified.

Moreover, the high transduction efficiency of SeV with a minimal amount of virus makes SeV well-suited for genome-wide genetic screening, where a low multiplicity of infection per cell is essential [80].

There are several limitations to our study. SeV was first isolated from mice. Although the SeV is safe for humans and non-human primates, SeV can infect rodents and swine; thus, it cannot be used for murine PDAC models in vivo. Toxicity is observed in some PDAC cell lines at higher SeV concentrations, with a slight correlation to initial cell viability and varying effects depending on the cell lines. Notably, PDX-derived primary PDAC cells do not show SeV toxicity.

Further investigation is needed to determine the extent of toxicity in both in vivo and in vitro settings for PDAC cells and other cell types.

We have tested limited AAV serotypes and cannot exclude the possibility that any other serotype AAV could give better transduction efficiency for PDAC or pancreatic ductal cells. However, due to the drawbacks of AAV vectors described above, using AAV vectors was not ideal for our study, and we haven't further explored all other AAV serotypes in detail.

Another limitation of our work is that we tested transduction efficiency for all cells in the monolayer condition. Since the possibility of variation due to the 3D culture condition cannot be ruled out, further study is needed to ascertain the applicability of these results to 3D culture settings, such as organoid culture.

## 5 CONCLUSIONS

Herein, we demonstrate using the SeV-vector for gene delivery in human normal and cancer pancreatic ductal cells in vitro as a proof-of-principle. Future investigation is needed to identify cell types SeV can effectively target in vitro and in vivo, extending beyond pancreatic ductal cells. Additionally, for in vivo cell therapy, such as direct reprogramming, further study is required to determine whether SeV-vector can effectively deliver multiple genes in a single cassette in a controllable manner in non-human primate settings in vivo.

## Funding

The work was funded by a 10.13039/501100000289CRUK-10.13039/100006668OHSU Project Award (C65925/A26986 to JK and AS), 10.13039/501100000403MRF New Investigator Grant (GCNCR1042A to JK), 10.13039/501100004919King Abdulaziz City for Science and Technology Health Sector (1078107040 to AA), and Knight 10.13039/501100011032CEDAR (68182-933-000, 68182-939-000) from the Cancer Early Detection Advanced Research Center at 10.13039/100006668OHSU Knight Cancer Institute.

## Ethics approval and consent participate

The current research was approved by the Institutional Review Board of the Oregon Pancreas Tissue Registry study (IRB00003609), and all patients signed an informed consent form.

## CRediT authorship contribution statement

**Dmytro Grygoryev:** Writing – review & editing, Writing – original draft, Visualization, Validation, Methodology, Investigation, Funding acquisition, Formal analysis, Data curation, Conceptualization. **Taelor Ekstrom:** Writing – review & editing, Validation, Methodology, Investigation. **Elise Manalo:** Resources, Methodology, Investigation. **Jason M. Link:** Resources, Methodology. **Amani Alshaikh:** Resources, Methodology. **Dove Keith:** Resources, Methodology. **Brittany L. Allen-Petersen:** Resources. **Brett Sheppard:** Resources. **Terry Morgan:** Writing – review & editing, Resources, Data curation. **Abdenour Soufi:** Resources, Methodology. **Rosalie C. Sears:** Supervision, Resources. **Jungsun Kim:** Writing – review & editing, Writing – original draft, Supervision, Project administration, Investigation, Funding acquisition, Formal analysis, Data curation, Conceptualization.

## Declaration of competing interest

The authors declare that they have no known competing financial interests or personal relationships that could have appeared to influence the work reported in this paper.
