# Supplementary material for: Sendai virus is robust and consistent in delivering genes into human pancreatic cancer cells
Source: Heliyon. 2024 Feb 28;10(5):e27221. doi: 10.1016/j.heliyon.2024.e27221 (PMC10923719; doi:10.1016/j.heliyon.2024.e27221)
Supplement: Multimedia component 1 [file mmc1.pdf]

Grygoryev & Kim et al., 2023

Figure S1

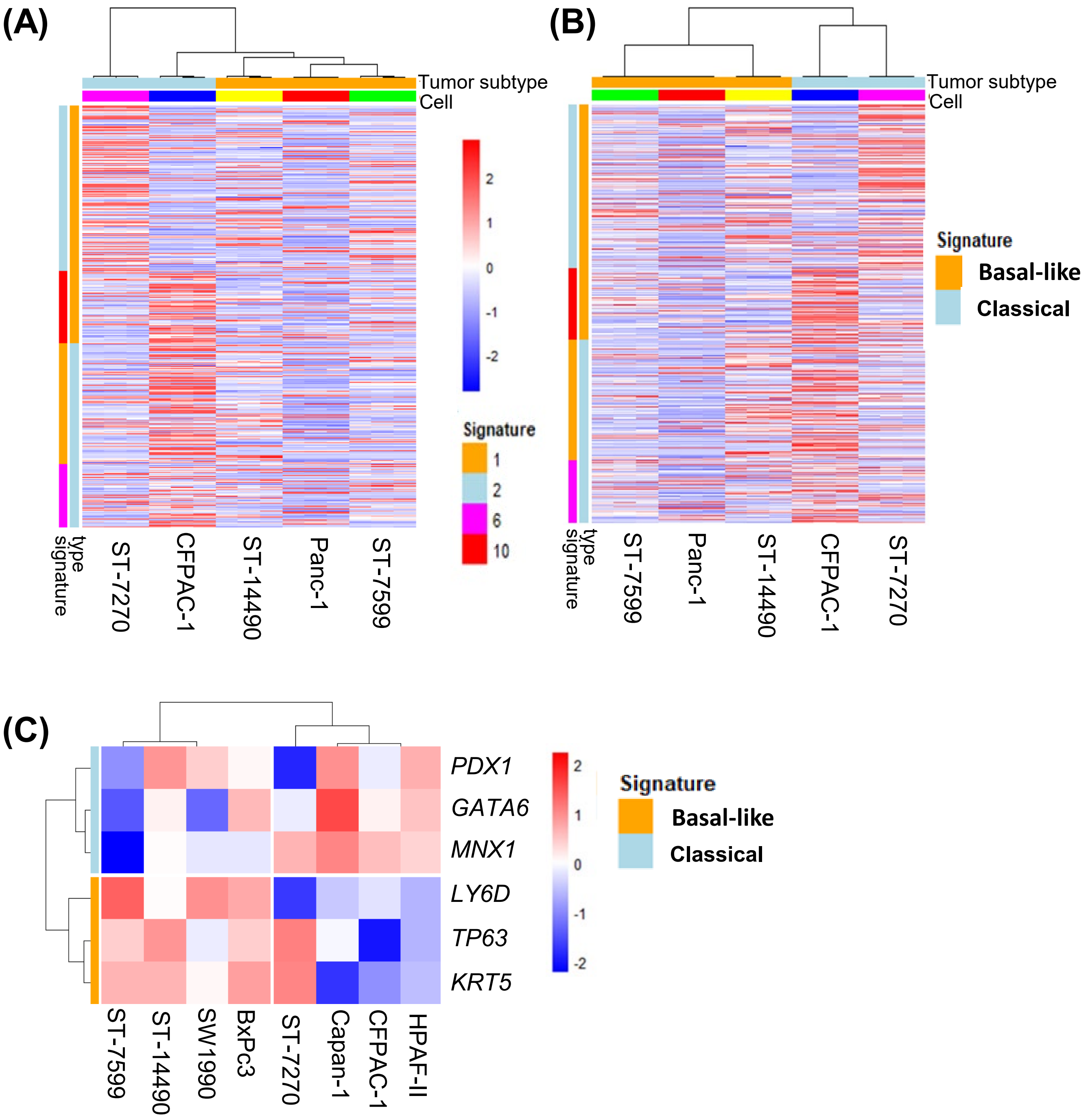

Grygoryev & Kim et al., 2023

Figure S2

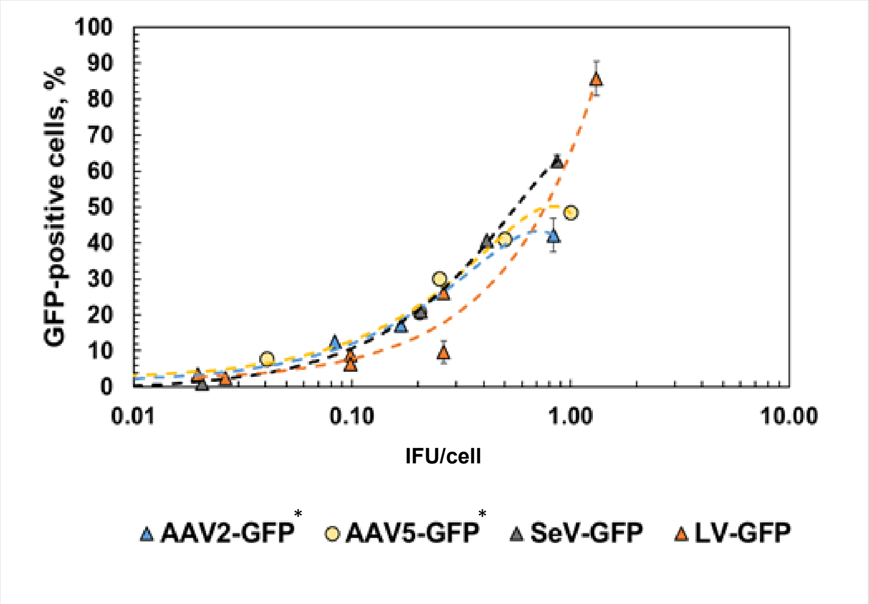

Grygoryev & Kim et al., 2023

Figure S3

(A)

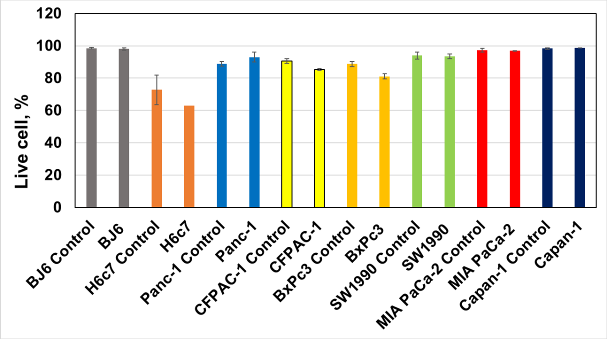

(B)

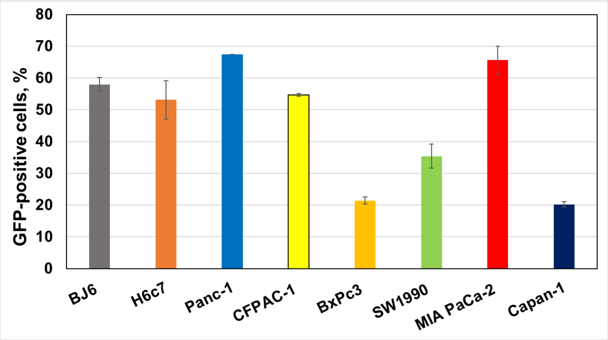

(C)

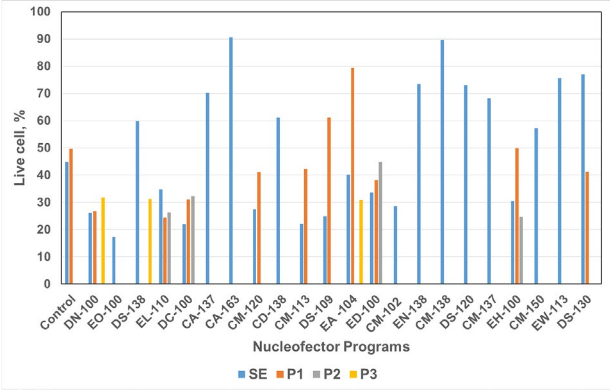

(D)

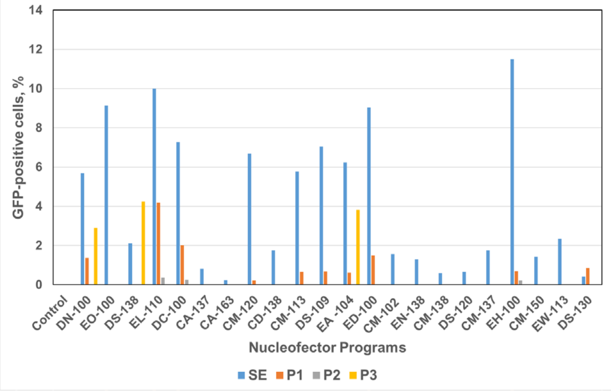

(E)

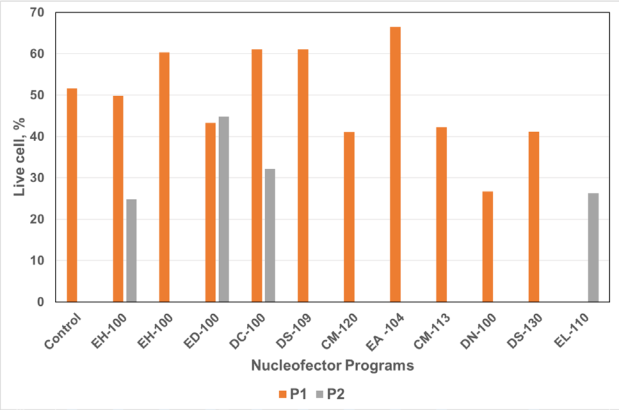

(F)

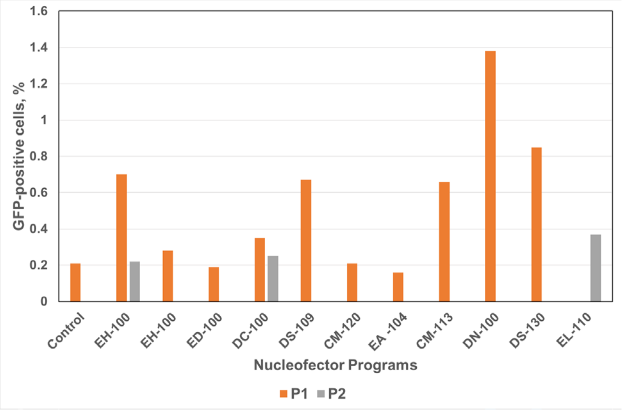

(G)

Cell	BJ6	H6c7	Panc-1	BxPc3	MIA PaCa-2	Capan-1	CFPAC-1	SW1990
Nucleofection program	FF-120	EO-100	DN-100	DN-100	DN-100	CA-163	CA-163	CD-138

Figure S1. (Related to the overall study) Comparison of subtypes of PDX-derived primary PDAC cells and PDAC cell lines. (A-B) Heatmaps of hierarchical (A) and consensus (B) clustering of normalized gene expression of Panc-1 and CFPAC-1 cell lines and primary PDAC cells ST-7599, ST-14490 and ST-7270 from PDX using PDAC subtype-gene signature[7]. The heatmap indicates the Z-score expression. (C) A heatmap of consensus clustering of six PDAC subtype marker genes in basal-like and classical PDAC subtypes of cells. The expression levels of six marker genes were obtained by qRT-PCR, normalized to the *GAPDH* level, and log 2 transformed (log 2-fold change values).

Figure S2. (Related to AAV methods in the result) Transduction efficiencies of LV-GFP, SeV-GFP, AAV2-GFP and AAV5-GFP in H6c7 - Normal pancreatic ductal epithelial cell. IFU/cell - Functional infectious unit per cell calculated based on corresponding viral vector titer in H6c7 cell (Table S1). The two tested AAV serotypes have similar transduction efficiencies (*p-value =0.187*) that are significantly lower than that of SeV (*p-value < 0.05*). P-values (ANOVA test) show the significance of the interaction of categorical variables (viral vector types) in the regression models describing the dependences of the fraction of GFP-expressing cells to the number of viral IFU/cell. The asterisks in the figure refer to the P-value of the ANOVA test. * - P ≤ 0.05; ** - P ≤ 0.01; *** - P ≤ 0.001; **** - P ≤ 0.0001. All data points on the figures have SD bars unless specified. SD bars can be smaller than the symbols' size.

Figure S3. (Related to the non-viral methods in the result) Comparison of nucleofection efficiencies in human normal cell lines, PDAC cell lines, and primary PDAC cells. (A-B) Comparison of the cell viability (A) and the transfection efficiency represented as the GFP expression (B) in normal control cell lines (BJ6, H6c7) and PDAC cell lines after nucleofection. (C-D) Effect of nucleofection conditions on the cell viability (C) and the transfection efficiency (D) of primary PDAC cells (ST-7599), where SE, P1, P2, and P3 nucleofector solutions and various nucleofector programs were used. (E-F) Effect of nucleofection conditions on the cell viability (E) and the transfection efficiency (F) of primary PDAC cells (ST-12908), where P1 and P2 nucleofector solutions and several nucleofector programs, which showed the highest efficiency in primary PDAC, were used. (G) The nucleofector programs used for nucleofection of control cells (BJ6 and H6c7) and PDAC cell lines are described in Fig. S4A-B. SD's are not presented for ST-7599 and ST-12908 primary PDAC cells (C-F) since only one measurement was made for each sample and condition.

Table S1. (Related to the overall study) Functional titers of Sendai viral GFP vector (SeV-GFP), lentiviral GFP vector (LV-GFP), adeno-associated serotype 5 viral GFP vector (AAV5-GFP) and adeno-associated serotype2 viral GFP vector (AAV2-GFP) in different cells.

Cell	SeV-GFP	LV-GFP	AAV5-GFP	AAV2-GFP
HT1080	$2.3x10^7 \pm 2.7x10^6$	$4.9x10^8 \pm 1.3x10^8$		
LLCMK2	$1.2x10^8$ *			
BJ6	$2.7x10^7 \pm 2.1x10^6$	$3.9x10^8 \pm 3.5x10^7$		
H6c7	$1.3x10^7 \pm 7.4x10^5$	$9.8x10^8 \pm 5.1x10^7$	$1.5x10^7 \pm 4.2x10^5$	$2.1x10^8 \pm 5.1x10^6$
Panc-1	$2.6x10^7 \pm 5.6x10^6$	$1.9x10^7 \pm 1.1x10^6$		
CFPAC-1	$2.3x10^7 \pm 4.8x10^6$	$3.8x10^6 \pm 7.7x10^5$		
SW1990	$1.7x10^7 \pm 3.1x10^6$	$1.8x10^7 \pm 3.1x10^6$		
BxPc3	$1.5x10^7 \pm 1.9x10^6$	$1.8x10^7 \pm 3.3x10^6$		
Capan-1	$1.7x10^7 \pm 1.3x10^6$	$1.2x10^7 \pm 2.1x10^6$		
MIA PaCa-2	$6.0x10^6 \pm 1.0x10^6$	$6.2x10^7 \pm 6.8x10^5$		
HPAF-II	$1.3x10^7 \pm 1.0x10^6$	$6.9x10^6 \pm 8.9x10^5$		
ST-7599	$2.9x10^7 \pm 4.3x10^6$	$2.6x10^7 \pm 3.0x10^6$		
ST-14490	$3.8x10^7 \pm 1.3x10^7$	$2.8x10^7 \pm 2.7x10^6$		
ST-7270	$8.7x10^6 \pm 3.0x10^6$	$1.9x10^6 \pm 2.9x10^5$		

Table S2. (Related to Fig.2A-B and 3A-B) The P-value of linear regression model coefficients.

	LV-GFP	SeV-GFP
H6c7	0.25	0.33
BJ6	0.78	0.03
Panc-1	0.14	<0.05
CFPAC-1	0.07	0.01
SW1990	0.75	0.71
BxPc3	0.14	0.75
Capan-1	0.23	0.01
MIA PaCa-2	0.2	<0.05
HPAF-II	0.39	0.2
ST-7599	0.82	0.11
ST-7270	0.34	0.22
ST-14490	0.4	0.48

Table S3. (Related to Fig.2C-D and 3C-D) Comparison of transduction efficiencies of viral vectors in normal and PDAC cells.

	p-value			
	LV-GFP		SeV-GFP	
	H6c7	BJ	H6c7	BJ
Panc-1	4.92E-05	4.38E-05	7.36E-02	2.14E-01
SW1990	3.31E-05	1.29E-05	6.52E-01	7.36E-03
BxPC3	3.34E-05	1.02E-05	6.52E-01	4.26E-03
MIA Paca-2	9.13E-03	4.75E-01	4.03E-03	2.35E-04
CFPAC-1	4.32E-09	9.81E-10	4.04E-02	6.95E-02
Capan-1	5.59E-07	6.04E-05	2.20E-02	1.33E-04
HPAF-II	7.64E-06	5.52E-07	8.15E-03	2.17E-04
ST-7599	1.57E-05	2.31E-03	4.99E-03	1.28E-02
ST-7270	1.87E-08	1.21E-09	2.64E-02	5.97E-04
ST-14490	1.87E-08	5.84E-01	1.52E-03	5.86E-03

Table S4. (Related to Fig. 4) Comparison of transduction efficiencies of LV-GFP and SeV-GFP vectors in normal and PDAC cells.

	p-value
H6c7	0.384
BJ6	0.04
Panc-1	0.093
SW1990	0.014
BxPC3	0.013
MIA Paca-2	0.482
CFPAC-1	2.65E-07
Capan-1	0.007
HPAF-II	0.055
ST-7599	0.002
ST-7270	2.82E-04
ST-14490	0.024

SUPPLEMENTAL TABLE LEGENDS

Table S1. (Related to the overall study) Functional titers of Sendai viral GFP vector (SeV-GFP), lentiviral GFP vector (LV-GFP), adeno-associated serotype 5 viral GFP vector (AAV5-GFP) and adeno-associated serotype2 viral GFP vector (AAV2-GFP) in different cells. Functional titers were determined by flow cytometry based on the percentage of transduced cells expressing GFP and presented as infectious units per milliliter (IFU/ml). * SeV-GFP titers in LLCMK2 were reported by the manufacturer without SD.

Table S2. (Related to Fig.2A-B and 3A-B) The *P-value* of linear regression model coefficients. The dependence of cell viability on the number of viral IFU/cells was fitted with a linear regression model. A linear regression model with logarithmic transformations was used for Panc-1 and MIA PaCa-2 cells transduced with SeV-GFP vectors. *P-values* show if regression coefficients are significantly different from zero. LV-GFP - lentiviral GFP vector; SeV-GFP- Sendai virus GFP vector. IFU/cell - Functional infectious units per cell calculated based on viral vector titer in HT1080 cell for LV-GFP and LLCMK2 for SeV-GFP (Table S1).

Table S3. (Related to Fig.2C-D and 3C-D) Comparison of transduction efficiencies of viral vectors in normal and PDAC cells. The dependence of the fraction of GFP-expressing cells on the number of viral IFU/cells used is fitted with a second-degree polynomial regression model. *P*-values (*ANOVA* test) show the significance of the interactions of categorical variables (cell type) in the regression models describing the dependences of the fraction of GFP expressing cells to the number of viral IFU/cell used in control (H6c7 and BJ6) and PDAC cells. LV-GFP - lentiviral GFP vector; SeV-GFP- Sendai virus GFP vector. IFU/cell - Functional infectious units per cell calculated based on viral vector titer in HT1080 cells for LV-GFP and LLCMK2 for SeV-GFP (Table S1).

Table S4. (Related to Fig. 4) Comparison of transduction efficiencies of LV-GFP and SeV-GFP vectors in normal and PDAC cells. The dependence of the fraction of GFP-expressing cells on the number of viral IFU/cell used was fitted with a second-degree polynomial regression model. *P*-values (*ANOVA* test) show the significance of the interaction of categorical variables (viral vector type) in the regression models describing the dependences of the fraction of GFP expressing cells to the number of viral IFU/cell used for LV-GFP and SeV-GFP vectors. IFU/cell

- Functional infectious units per cell calculated based on viral vectors' titer in corresponding cells (Table S1).
